# Research highlights, *The Plant Genome*, Volume 16, Issue 3

**DOI:** 10.1002/tpg2.20386

**Published:** 2023-08-31

## 1 TRANSCRIPTOMICS UNCOVERS FUSARIUM WILT RESISTANCE IN CHICKPEA

Fusarium wilt (FW), caused by *Fusarium oxysporum* f. sp. ciceris (Foc), is a devastating soilborne disease that causes significant yield losses in chickpea worldwide. Management of FW through crop rotation strategies or chemical control is challenging, and thus, resistance breeding is the most cost‐effective and environmentally sustainable strategy. In this study, Garg et al. (https://doi.org/10.1002/tpg2.20340) employed a transcriptomics approach to investigate the molecular mechanisms of FW resistance in chickpea. The analysis of different samples representing contrasting resistance sources of chickpea revealed 5182 differentially expressed genes (DEGs), involved in various biological processes such as defense response, cell wall biogenesis, secondary metabolism, and disease resistance. Further, the study identified DEGs that co‐localized with previously reported quantitative trait loci for FW resistance and several resistance/susceptibility‐related genes exhibiting contrasting expression patterns in resistant and susceptible genotypes. These findings represent a significant contribution to our understanding of the molecular mechanisms underlying FW resistance in chickpea and can aid in the development of new chickpea cultivars with enhanced FW resistance.

## 2 META‐ANALYSIS SHEDS LIGHT ON TRAITS GENETICS IN PIGEONPEA

The identification of the most promising genomic regions and markers significantly associated with key traits is a major challenge for pigeonpea molecular breeding programs. Halladakeri et al. (https://doi.org/10.1002/tpg2.20342) conducted a meta‐analysis of quantitative trait loci associated with agronomic traits, fertility restoration, disease resistance, and seed quality traits in pigeonpea. The study identified several major genomic regions that were found significantly associated with the target traits. Furthermore, putative candidate genes underlying some of these regions were also identified. Synteny and collinearity analyses among pigeonpea and four related legume crops were also conducted, leading to the identification of numerous conserved genomic regions. The markers associated with the major genomic regions have the potential to be utilized for molecular breeding and genomic selection, while the candidate genes can serve as targets for functional analysis to elucidate the underlying molecular mechanisms.

## 3 ASSEMBLY AND ANALYSIS OF *Melilotus officinalis* GENOME

The unknown genome of *Melilotus officinalis* restricted the domestication and utilization of the species and its germplasm resource diversity. He et al. (https://doi.org/10.1002/tpg2.20345) assembled and analyzed a chromosome‐scale assembly of the *M. officinalis* genome. The 976.27 Mb of genome was divided into eight chromosomes, covering 99.16% of the whole genome. A total of 50,022 genes were predicted in the genome. *M. officinalis* and *Melilotus albus* shared a common ancestor 0.5–5.65 million years ago (MYA). A genome‐wide doubling event occurred 68.93 MYA according to the synonymous nucleotide‐substitution values. A total of 552,102 tandem repeats (TRs) were predicted, and 46,004 SSR primers of TRs with 10 or more base pairs were developed and designed. The elucidation of the *M. officinalis* genome provides a compelling model system for studying the genetics, evolution, and biosynthesis of this legume.

## 4 MOBILE ELEMENTS TRANSPOSE IN WHEAT GENOME

Bread wheat has a complex genome shaped by millions of repeated copies of transposable elements (TEs). Their evolutionary dynamics and contribution to wheat genomic variability were still largely unexplored because of the complexity of such analyses. Papon et al. (https://doi.org/10.1002/tpg2.20347) established a dedicated bioinformatics approach to accurately compare 17 assembled genome sequences of cultivated wheat and wild *Triticeae* relatives. TE insertions and deletions were likely balanced across the *Triticeae* evolution, and traces of recent transpositions were detected for almost all TE families. Each family tends to maintain a constant rate of transposition, meaning that TEs did not recently amplify by burst and that polyploidization did not trigger any transposition boost, as previously thought. Conclusions support an equilibrium model of evolution, suggesting TEs are important structural genome components.

## 5 ANTIMICROBIAL PEPTIDES IN *Dendrobium officinale*

*Dendrobium officinale* is an endangered *Orchidaceae* herb in China. Huang et al. (https://doi.org/10.1002/tpg2.20348) report that *Dendrobium officinale* has diverse types of antimicrobial peptides (AMPs) that enhance the plant's innate immunity and disease‐resistance capability. Gene codon usage analysis of these antimicrobial peptides revealed a weak usage bias, and the preference was mainly influenced by natural selection pressure. Through peptide structural and domain analyses, some typical antimicrobial domains were identified in *D. officinale* AMPs. Gene expression patterns of AMPs were also investigated under various abiotic and biotic stresses. *Dendrobium officinale* AMPs can be induced by salt dress, while drought stress did not show the same impact. Moreover, salicylic acid and jasmonic acid signaling pathways might be involved in most of the AMPs expressions.

## 6 WILD STINKING GOOSEFOOT GIVES INSIGHTS INTO QUINOA'S ORIGINS

The Andean superfood quinoa has complex origins, tracing its way back to North America. Young et al. (https://doi.org/10.1002/tpg2.20349) report the whole genome sequence of a wild plant from the Southwest, Watson's stinking goosefoot, and used that sequence to compare the genomes of over 40 wild and cultivated goosefoot strains to identify quinoa's most likely North American ancestor. Their study showed that a very rare plant from the arid interior of western North America, sandhill goosefoot, may have anciently donated its set of 18 chromosomes to the genome of quinoa's original 36‐chromosome wild ancestor. This information should help guide breeders as they access wild species for improving quinoa's productivity in adverse environments.

## 7 GENOMIC PREDICTION OF SCAB RESISTANCE

*Fusarium* head blight, also known as scab, is a devastating disease of wheat that causes the formation of mycotoxins, and the Southern Uniform Winter Wheat Scab Nursery is a public resource for testing the *Fusarium* head blight resistance of late development lines. Winn et al. (https://doi.org/10.1002/tpg2.20353) illustrated the power of this public resource by pairing the phenotypic data in the Scab Nursery with existing genomic sequencing data to produce genomic predictions of early development lines. These findings demonstrate that a public resource, like the Scab Nursery, should be used in training genomic prediction models and that there should be a regional data coordinator of these nurseries to provide resources for genomic prediction of early development lines in collaborating public wheat breeding programs.

## 8 PEST‐RESISTANT BEANS FOR THE FUTURE

Tepary beans are not as widely grown compared to common beans but show extreme resilience to insects and diseases. Bornowski et al. (https://doi.org/10.1002/tpg2.20363) analyzed sequences from hundreds of tepary lines to associate genomic regions associated with resistance to key pests. Candidate resistance genes in the tepary genome were identified that provided protection against insects, bacteria, viruses, and other agronomic pests. Additionally, tepary diversity was highlighted to reveal subpopulations among cultivated and wild lines. Promising tepary lines with resistance to key pests and pathogens will facilitate future breeding and improvement of this hardy crop.

## 9 CELL WALLS MODIFY BEAN COOKING TIMES

Dry beans are a nutritious food that often have long and highly variable cooking times. Pre‐soaking is one of the major methods used by consumers to reduce cooking times. Jeffery et al. (https://doi.org/10.1002/tpg2.20364) used gene expression analysis to understand molecular changes that occur during soaking in dry bean genotypes with different cooking times. After 12 h of soaking, the faster cooking beans took 18–19 min to completely cook, whereas the slower cooking beans took 23.5–30 min. The cooking times of all four bean genotypes studied decreased drastically from 0 to 12 h of soaking and remained stable after 12 h. Nine gene co‐expression pathways were significantly associated with changes in both soaking time and cooking time for the beans. Upon further investigation, genes in these pathways related to cell wall growth and development as well as hypoxic stress were more expressed in the slower cooking dry beans during soaking. This suggested that water uptake triggers cell wall thickening/strengthening in slower cooking bean varieties, leading to elongated cooking times.

## 10 THE CONSERVATION OF GENE MODELS CAN SUPPORT GENOME ANNOTATION

While genome sequencing and assembly becomes relatively easy, the ability to predict accurate gene models from the sequence remains a challenge. Researchers need to balance the ability to predict all real genes while avoiding predicting false genes. Here, Fernandez et al. (https://doi.org/10.1002/tpg2.20377) have developed a method to help validate predicted gene models based on the conservation of gene sequences across related species. They developed a set of 15,345 representative gene models from 12 legume assemblies that can be used to support genome annotations for other legumes. If a new gene model does not match the representative set, then it is likely to be a false model and further evidence would be required to include it in the annotation. Representative sets can be generated for any group of species and used to support confident gene predictions for the rapidly growing number of genome assemblies.

